# Difference in coagulation systems of large animal species used in cardiovascular research: a systematic review

**DOI:** 10.1007/s10047-024-01446-y

**Published:** 2024-05-20

**Authors:** Louis Staelens, Tom Langenaeken, Filip Rega, Bart Meuris

**Affiliations:** Department of Cardiovascular Diseases, Research Unit of Cardiac Surgery, University Hospitals Leuven, Herestraat 49, 3000 Leuven, Belgium

**Keywords:** Cardiovascular system, Blood coagulation, Sheep, Swine, Heart valve prosthesis

## Abstract

Preclinical testing using animal models is indispensable in cardiovascular research. However, the translation to clinical practice of these animal models is questionable since it is not always clear how representative they are. This systematic review intends to summarize the interspecies differences in the coagulation profile of animal models used in cardiovascular research. It aims to guide future research in choosing the optimal animal species. A literature search of PubMed, Embase, Web of Science (Core Collection) and Cochrane Library was performed using a search string that was well defined and not modified during the study. An overview of the search terms used in each database can be found in the appendix. Articles describing coagulation systems in large animals were included. We identified 30 eligible studies of which 15 were included. Compared to humans, sheep demonstrated a less active external pathway of coagulation. Sheep had a higher platelet count but the platelet activatability and response to biomaterials were lower. Both sheep and pigs displayed no big differences in the internal coagulation system compared to humans. Pigs showed results very similar to those of humans, with the exception of a higher platelet count and stronger platelet aggregation in pigs. Coagulation profiles of different species used for preclinical testing show strong variation. Adequate knowledge of these differences is key in the selection of the appropriate species for preclinical cardiovascular research. Future thrombogenicity research should compare sheep to pig in an identical experimental setup.

## Introduction

As stated by the WHO, cardiovascular diseases (CVDs) are accountable for 32% of all deaths worldwide which makes it the leading cause of death [1]. More than four out of five cardiovascular deaths are because of cardiac attacks and strokes, of which a third occur prematurely in people under the age of 70. CVD consists of coronary heart disease, cerebrovascular disease, peripheral arterial disease, rheumatic heart disease, congenital heart disease, deep vein thrombosis, and pulmonary embolism. These dysfunctions are usually caused by a problem in hemostasis resulting in thrombosis or bleeding [1].

In current cardiovascular research and development of cardiovascular devices, preclinical testing on animal models is crucial to evaluate the safety and feasibility of these interventions before human trials. The most used animals are sheep and pigs and in some cases calves or goats. These animal experiments are helpful because they have a similar coagulation compared to humans. However, in some cases, the animal model will not be able to provide relevant information about the efficacy of the product [2]. This mismatch between animal experiments and clinical trials has been reported and may be due to biological differences rendering the animal models inadequate to represent human complications [2]. The purpose of this review is to compare the thrombogenicity and coagulation of sheep and pigs to evaluate which animal is best fitting to be used in cardiovascular research.

## Methods

### Data collection

**Fig. 1:**
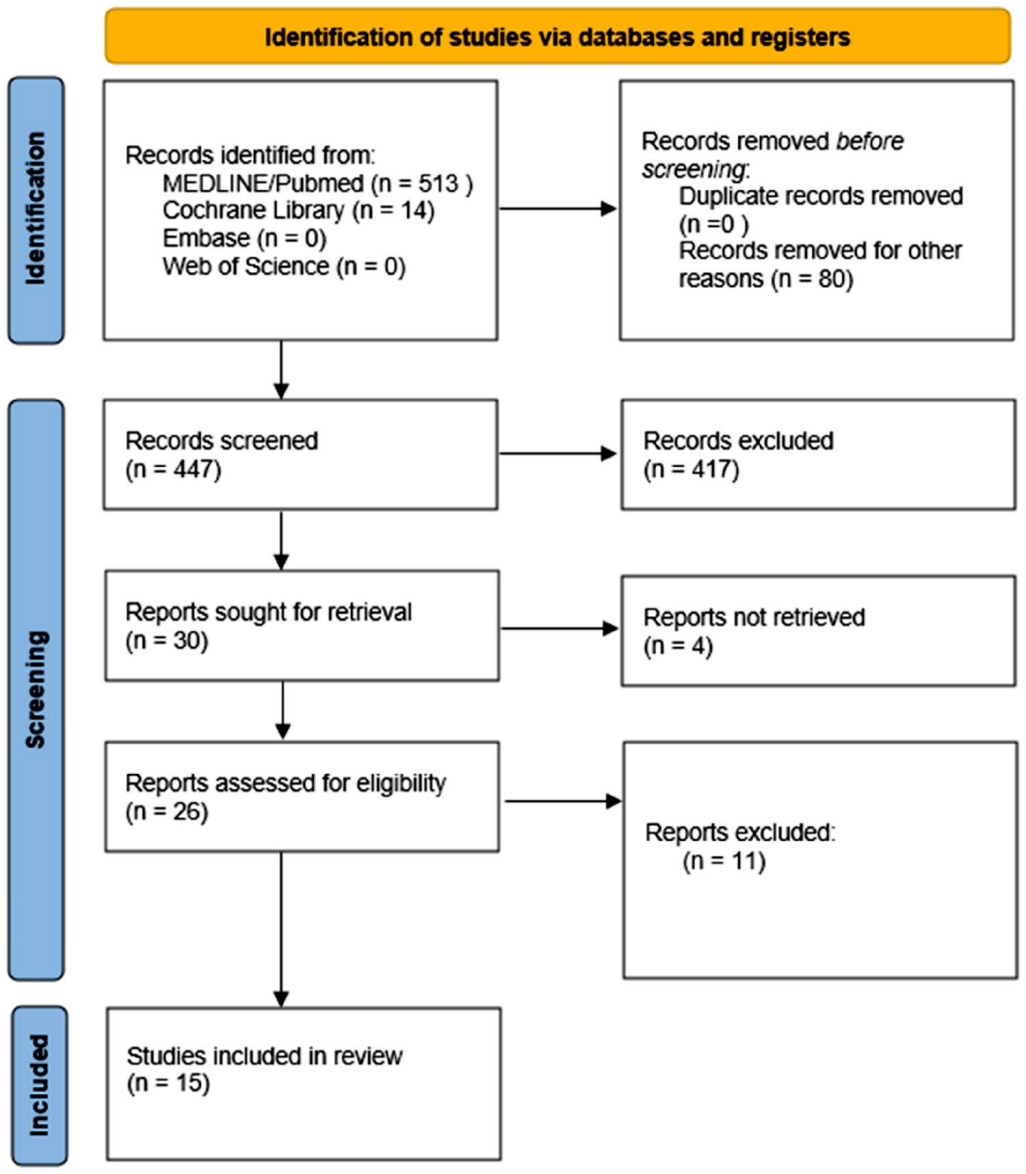
PRISMA flow diagram 1

This systematic review is intended to summarize the most recent data on the topics “cardiovascular research” and “large animals”. It gives an overview of in vitro and in vivo evidence of the animal models currently used in cardiovascular research. This analysis was conducted in accordance with current guidelines for performing systematic reviews by following the preferred reporting items for systematic reviews and meta-analyses (PRISMA) guidelines, as can be seen in Fig. 1. We performed a systematic review of data from inception to April 2023 through the PubMed/Medline database, Embase, Web of science (core collection) and Cochrane Library with no language limitations. The following search string was used: (“cardiac” or “cardiovascular”) and (“blood coagulation"[mesh] or "coagulation cascade" or "coagulation" or “platelets”) and (“sheep” or “pig” or “pigs” or “preclinical testing”) not (“xenotransplantation”).

We searched for original data in both in vivo and in vitro experiments in pigs, sheep, and humans. We explored relevant papers and screened for studies researching coagulation in large animals and humans.

### Study inclusion

Potentially eligible studies were identified by examination of the article title. A screening and selection of abstracts was conducted after the detection and removal of duplicates. Full articles were obtained for eligibility assessment when a study was deemed relevant. Studies were selected by 2 independent reviewers. The inclusion of studies was decided unanimously.

## Results

### Study selection

Our search strategy generated a total of 527 results from all the different databases listed above. No duplicates were found. We exported these articles to Rayyan, in which 2 people independently screened the studies by title and abstract. There were 30 articles selected for full-text eligibility, from which 15 articles finally remained to be included in our review (Fig. 1). Out of our 15 articles, 12 discuss the cardiovascular system of humans. Eight of these 12 articles look at sheep and 8 evaluate pigs. The remaining 3 articles describe coagulation only in pigs. All of these studies are summarized in Tables 1 and 2.

**Table 1:** Overview of blood values of humans, sheep and pig

Blood value	Humans	Sheep	Pigs
Platelets (× 10^3^/ml)	218–286 [4] 150.00–450.00 [3] 140–400 [5] 187 [6] 261 ± 47 [8] 243 ± 33 [14]	470 (462–485) [4] 342.02–535.82 [3] 401 (± 144) [5] 742 [6] 475 [8]	430 [6] 305 ± 67 [7] Higher than humans [14] 343 ± 88 [15] 235 ± 29 [16] 316.7 ± 33.7 [17]
Hematocrit (%)	41.50–50.40 [3] 33–47 [5] 42 ± 4.0 [14]	26.22–28.82 [3] 28 (± 3) [5]	40 ± 3.4 [7] 32.7 ± 3.2% [15] 29.2 ± 0.7% [17]
Fibrinogen (g/l)	3.0 (2.8–3.6) [4] 1.80–3.50 [3] 2–4.5 [5] 1.80–3.90 [6]	2.2 (1.7–3.3) [4] 1.78–2.15 [3] 2.61 (± 0.73) [5] Comparable to human [6]	Comparable to human [6] 170 ± 47 mg/dl [16] 185.8 ± 15.5 mg/dl [17]
vWF (IU/dl)	50.00–150.00 [3]	101.5–118.05 [3] 117.1 (± 34.9)% of human concentration [5]	N/A
Factor VII	N/A	13% of human concentration [4]	N/A
Factor VIII	N/A	816.9 (± 201.4)% of human concentration [5]	N/A
Antithrombin (IU/dl)	70.00–120.00% of the reference value [3]	Lower than human [4] 65.77–72.72 [3] 93.0 (± 8.5)% of human concentration [5]	N/A
Protein C	N/A	Lower than human [4] 48.6 (± 9.3)% of human concentration [5]	N/A
ETP (nM·min)	4235 [6]	4092 [6]	2043 [6]
PT (s)	63.5 ± 11.8 [2] 14.8 (14.2–16) [4] 70.00–130.00% of the reference value [3] 9–13 [5] 21 [6]	12.3 (11.7–13.7) [4] 52.26–57.2% of the human value [3] 13.10 (± 1.1) [5] 40 [6]	23 [6] 9.2 ± 0.7 [16]
aPTT (s)	192.5 ± 29.0 [2] 29.1 (28.1–30.1) [4] 26.00–35.00 [3] 24–39 [5]	26.2 (23.9–30.5) [4] 30.74–35.26 [3] 29.00 (± 5.78) [5] Comparable to human [6]	Comparable to human [6] 17 ± 0.6 [15] 11.0 ± 0.7 [16]
CT (s)	103.1 ± 7.1 [2] 372 (360–384) [4] 137–246 [5] 595 (476–901) [6] 1290.8 [8]	264 (240–282) [4] 68–210 [5] 494 (344–1431) [6] 1163.3 ± 206.5 [8]	116.7 ± 10.5 [2] 244 (146–296) [6] 110 ± 8 [15] 41 ± 4 [16]
CFT (s)	72 (66–72) [4] 40–100 [5] 200 (104–436) [6]	1.1 (0.1–1.1) [4] 35–127 [5] 182 (143–532) [6]	52 (30–84) [6] 47 ± 8 [16]
CT + CFT (s)	733 (642–1197) [6]	685 (503–1963) [6]	298 (176–349) [6]
α-angle (°)	71.4 (70.8–72.8) [4] 71–82 [5]	75.1 (73.9–76.9) [4] 69–83 [5] Comparable (64.18°) to human [10]	Comparable to human [2] 81 ± 2 [16]
MCF (mm)	59.1 ± 6.0 [2] 70.1 (70.2–73.9) [4] 58 (49–65) [6]	77.1 (75.4–80.5) [4] 72 (61–77) [6] 68.6 [10]	Higher than human [2] 74 (68–79) [6] 70 ± 4 [16]
MCF-*t* (s)	N/A	Similar to human [4] Shorter than human [10]	Shorter than human [2]
ML (%)	21 (2–24) [6]	2 (0–26) [6]	17 (12–31) [6]
PLT activation by glass	N/A	Comparable to human [12]	Comparable to human [20]
PLT activation by ADP (ohm)	11.81 ± 4.76 [8] 63–77% of the platelets [14]	Higher than human [4]	48–63% of the platelets [7]
PLT activation by collagen (ohm)	30.93 ± 6.31 [8] 80–90% of the platelets [14]	20.22 ± 7.30 [8]	80–90% of the platelets [7]
Fibrinolysis	N/A	Lower than human [4]	Similar to human [6]

*vWF* von Willebrand factor; *ETP* endogenous thrombin potential; *PT* prothrombin time; *aPTT* activated partial thromboplastin time; *CT* clotting time; *CFT* clot formation time; *CT + CFT* sum of the clotting time and the clot formation time; *MCF* maximum clot firmness; *MCF-t* time to MCF. *ML* median maximum lysis. *PLT*: platelet; *ADP* adenosine diphosphate

**Table 2:** Summarization of the articles included in this review

References	Background	Methods	Results	Conclusion
Mizuno et al. [2]	• Relevance of animal models to human health is often not clear • Purpose of this study: compare the clotting parameters of animal models to determine which animals most adequately mimic human clotting parameters	• The clotting parameters of humans, calves, goats and pigs were assessed in whole blood by in vitro thromboelastography using the clotting activators, such as tissue factor and partial thromboplastin phospholipid	• The maximum clot firmness (MCF) in domestic animals was significantly higher than that of humans	• There are relevant differences in the four species’ extrinsic and intrinsic clotting parameters. This indicates that it is necessary to clarify the characteristics of clotting properties in preclinical animal studies
Gruzdeva et al. [4]	• Aim: compare the hemostasiological profile in sheep and patients with coronary heart disease to predict thrombotic risks during preclinical tests of vascular prostheses on a large laboratory animal model	• Measurement of functional activity of platelets, prothrombin activity, INR, aPTT, thrombin time, fibrinogen concentration, antithrombin III and protein C activity and fibrinolysis • Changes in clot formation and viscoelastic properties of clots were assessed	Sheep had compared to humans: • Increased platelet response to ADP, little response to epinephrine • Increased activity of the PT complex, shortened thrombin time • Comparable APTT and fibrinogen values • Decreased anticoagulation and fibrinolysis	• Hemostasiological profile of sheep is characterized by the increased speed of thrombus formation, greater strength of the formed clot, and lower lysis ability as compared to CHD patients
Wilhelmi et al. [3]	• Information about ovine blood reference values is scanty • Aim: establish a reference list of ovine blood parameters relevant for blood coagulation	• The following parameters were evaluated in 47 mature ewes: cells and cellular components, global tests of coagulation and parameters relevant to blood coagulation	Interspecies differences (sheep to human): • Higher ovine ranges for some cell counts (neutrophils, lymphocytes, basophils, eosinophils, and platelets) • Lower values for some other parameters (Hb, HCT, MCV, MCH, AT, and Quick’s test)	• A reference list of ovine blood count and blood coagulation parameters was established. Because of some peculiarities of the ovine blood, this list may have implications for the interpretation of experimental data
Foley et al. [5]	• Coagulation studies involving sheep are limited	In 50 healthy sheep and compared to corresponding human ranges: • Full blood examination • Routine and specialized coagulation tests • Rotational thromboelastometry • Whole blood platelet aggregometry	• Sheep had a similar PT, aPTT, TCT, Fib(C), vWF, AT, Plasmin Inh • High levels of FVIII, low Protein C, greater overall clot firmness and a reduced capacity for clot lysis was documented in sheep	• The application of a spectrum of coagulation assays has enabled elucidation of the similarities as well as differences between ovine and human coagulation
Siller-matula et al. [6]	• The relevance of animal models to human health is often questioned because of differences between species • Aim of this study: to find an appropriate animal species which mimics the coagulation profile in humans most adequately	• Rotation Thromboelastometry • Endogenous thrombin generation was measured in platelet-poor plasma • Measurements were performed in blood from five different species: humans, rats, pigs, sheep and rabbits	• In humans and sheep, the clotting time (ROTEM) with or without thrombin stimulation and endogenous thrombin potential (ETP) were in the same range	• Sheep had a clotting time most similar to humans and could thus be a suitable species for translational coagulation studies
Clauser et al. [7]	• Animal and particularly abattoir blood might present species-specific differences to human blood as well as elevated blood values, and pre-activated platelets due to stressed animals and non-standardized blood collection	Porcine abattoir blood in comparison to human donor blood: • Light transmission aggregometry • Aggregation kinetics of platelet rich plasma after stimulation with three different concentrations of each adenosine diphosphate (ADP) and collagen	• Activation with collagen revealed no significant differences in platelet behavior • Stimulation with ADP resulted in a lower maximum aggregation and a high disaggregation for porcine abattoir blood	• The similarities in platelet activation following collagen stimulation and the preservation of the porcine-specific reaction to ADP prove a general functionality of the porcine abattoir blood
Sato et al. [8]	• Differences in platelet function and coagulation systems between human and animal models have long been suggested but have not been well characterized, mainly because of methodologic limitations	Platelet and coagulation function were evaluated in bovine, ovine and canine species with a clot signature analyzer (CSA). Three CSA parameters were measured: • Platelet mediated hemostasis time (PHT) • Collagen induced thrombus formation time (CITF) • Clotting time (CT)	• Ovine and human blood showed similar PHT and CT values • CITF time values were significantly shorter in sheep • ADP induced platelet aggregation showed similar responsiveness in four species as did CITF	• These results may suggest that sheep are an acceptable animal model for testing the blood compatibility of devices
Johnson ca et al. [10]	• Tools to assess blood biocompatibility in the ovine model are limited and continued investigation to identify and apply additional assays is merited	• Thrombelastography was used to characterize normal ovine parameters • Platelet labeling with biotin was evaluated for its potential applicability to quantify ovine platelet life span	• Ovine reaction time was significantly shorter • Maximum amplitude, actual clot strength, and coagulation index were all significantly higher	• The collection of these parameters for normal ovines demonstrates the applicability of these techniques for subsequent studies where cardiovascular devices may be evaluated • This study provides an indication of normal ovine values for comparison purposes
Goodman et al. [11]	• Platelets play a major role in determining the hemocompatibility of mechanical heart valves and other high-shear-rate cardiovascular devices • Since larger animals are required to test many such devices, sheep and porcine platelet responses were compared to humans	Adhesion, spreading, and the formation of thrombus-like structures were examined in vitro on: • Pyrolytic carbon mechanical heart valve leaflets • National Institutes of Health-reference polyethylene • Silicone rubber • Formvar	• Porcine and human platelets spread extensively on pyrolytic carbon, formed thrombus-like structures on Formvar, and were least active on silicone rubber • Human platelets spread extensively on polyethylene, while porcine platelets remained pseudopodial • Sheep platelets attached much less, never reached fully spread shapes, and were far less active overall	• Since porcine responses were generally similar to humans, pigs may be a useful predictor of in vivo platelet—biomaterial interaction in humans • As ovine platelets were much less active, this must be accounted for in the evaluation of cardiovascular devices tested in sheep
Greif et al. [12]	• In sheep and pigs, medical products, developed and tested for human medical purposes, are almost exclusively used in interventional studies. Therefore, the extent to which platelets from human and ovine blood differ in terms of adherence, aggregation and activation after a 4–8 min exposure to glass was investigated	• Testing was performed with platelet-rich plasma (PRP) and a modified chandler loop-system, with 4–8 min blood-material exposure times corresponding to 20 and 40 test cycles, respectively, through the entire silicone tube loop of the test system	• Contact with the silicone tubing resulted in a decrease in platelet count in both species. Four more minutes caused a further decrease of the platelet count only in sheep PRP • When adding glass beads, these effects were more pronounced and stronger in sheep • With glass exposure in human and sheep PRP, the mean platelet volume increased, but this increase was stronger in sheep • Regarding activation, the activation markers CD62P and CD63 were detectable only in < 30% (sheep) and < 45% (human) of platelets, whereas after glass exposure, the proportion of CD62P + and CD63 + cells was more increased than before only in sheep	• Ovine platelets adhere more strongly to glass and show stronger aggregation behavior after glass contact than human platelets • Ovine and human platelets differ only slightly in activatability by glass
Krajewski et al. [14]	• One standard method for the investigation of platelet function under experimental conditions is flow cytometry • This approach is limited by a shortage of feasible antibodies and a lack of incubation protocols with regard to porcine platelets • This study aimed to establish a method for the investigation of porcine platelets in flow cytometry	• Platelets from pigs and human donors were stained with various commercially available specific antibodies against platelet receptors CD41a, CD42bα, CD62P, activated CD41/CD61, and platelet-bound fibrinogen • Platelet counts were determined and ADP-induced platelet aggregations of both species were examined to confirm that the agonist ADP reliably activates human as well as porcine platelets	• Five of the investigated antibodies bound to human, but not to porcine platelets • Two chicken-derived antibodies directed against CD62P and fibrinogen as well as a monoclonal mouse anti-CD62P and a polyclonal rabbit anti-fibrinogen antibody allowed reliable detection of porcine platelet activation	• This study describes a reliable method to detect the activation of porcine platelets and therefore provide a useful tool for platelet flow cytometry in porcine models • Notably, the applied incubation protocol and medium, in which platelets are suspended, have major effects on antibody-binding properties
Mueller et al. [15]	• The purpose of this study was to analyze whether standard human coagulation tests could be applied to calve and pig models in the setting of prolonged perfusion	• The thrombogenicity of membrane oxygenators as well as clotting parameters profiles, using standard human clotting tests, was analyzed in calves and pigs during 6 h perfusion • Blood samples were taken for coagulation variables throughout perfusion, and oxygenators were examined for clot deposits at the end of the experiment	• Baseline coagulation variables of pigs showed values similar to those of humans • Neither extrinsic nor intrinsic pathways could be activated in calves with standard human coagulation tests	• The pig model is a better model in which both coagulation pathways could be activated with standard human coagulation tests
Zentai et al. [16]	• Fibrinogen supplementation is currently being considered as a therapeutic option for hemostatic management of trauma related bleeding • This study investigated the impact of fibrinogen supplementation in a 24 h porcine model of blunt liver injury	• Coagulopathy was induced in 20 German land race pigs by hemodilution and blunt liver injury • Animals randomly received fibrinogen concentrate (100 mg/kg) or saline • Coagulation parameters were assessed and thromboelastometry (ROTEM) was performed	• Fibrinogen concentrate reduced the prolongations of EXTEM clotting time, EXTEM clot formation time, and prothrombin time induced by hemodilution and liver injury. A decrease in clot strength was also ameliorated • Endogenous thrombin potential was higher in the fibrinogen group than in the control group • Approximately 12 and 24 h after starting fibrinogen concentrate/saline infusion, all parameters measured in the 2 groups were comparable	• This study suggests that, in trauma, fibrinogen supplementation may shorten some measurements of the speed of coagulation initiation and produce a short-lived increase in endogenous thrombin potential, potentially through increased clotting substrate availability
Martini et al. [17]	• Hemorrhagic coagulopathy is involved in the morbidity and mortality of trauma patients. Nonetheless, many aspects of the mechanisms underlying this disorder are poorly understood • This study investigated changes in fibrinogen metabolism and coagulation function after a moderate hemorrhagic shock, using a new stable isotope approach	• Twelve pigs were randomly divided into the control (C) and hemorrhage (H) groups • Hemorrhage was induced by bleeding 35% total blood volume over a 30-min period • A primed constant infusion of [1-13C]phenylalanine (Phe), d5-phenylalanine, and α-[1-13C]- ketoisocaproate (KIC) was given to quantify fibrinogen synthesis and breakdown, together with measurements of circulating liver enzyme activities and coagulation function	• Fibrinogen fractional synthesis rate increased from 2.7 ± 0.2%/h in C to 4.2 ± 0.4%/h in H by Phe (*p* < 0.05) and from 3.1 ± 0.4%/h in C to 4.4 ± 0.5%/h in H by KIC (*p* < 0.05) • Fibrinogen fractional breakdown rate increased from 3.6 ± 1.0%/h in C to 12.9 ± 1.8%/h in H (*p* < 0.05) • The absolute breakdown rate accelerated from 3.0 ± 0.4 mg/kg^−1^ h^−1^ in C to 5.4 ± 0.6 mg/kg^−1^ h^−1^ in H (*p* < 0.05), but the absolute synthesis rate remained unchanged	• This study concludes that the observed changes in coagulation after hemorrhagic shock are mechanistically related to the acute acceleration of fibrinogen degradation
Greif et al. [20]	• Because most medical devices for cardiovascular interventional procedures are developed for humans they are tested mostly for compatibility with human blood • The aim of this study was to determine whether there are differences in behavior of porcine and human platelets when making contact with glass, which was used as an exemplary thrombogenic material	• Changes of platelet count, platelet volume and platelet expression of the activation markers CD61, CD62P and CD63 were measured using a modified chandler loop-system simulating the fluidic effects of the blood flow	• Minipig and human platelets showed significant differences in number and volume, but not in activation after 4–8 min exposure to glass	• Minipig and human platelets showed significant differences in number and volume, but not in activation after 4–8 min exposure to glass

### Interspecies coagulation differences

#### Humans

Human hematological reference values have been heavily researched, are widely known and used in all aspects of medicine. An overview of the discussed coagulation values can be found in Table 1. Wilhelmi et al. reported human platelets to be in the range of 150–450 × 10^3^/ml [3]. All platelet values found in other studies lay within this range with the exception of the results of Foley et al., who report a normal value of 140–400 × 10^3^/ml and so a slightly decreased lower limit of normal [4–8]. For blood hematocrit, there is an overlap between the results. Clauser et al. had a Hct of 42 ± 4.0% in their study, but mentioned a reference value of 37–50% [7]. This is about the same value as given by Wilhelmi et al. who observed a value of 41.50–50.40% [3]. Another study had a value of 33–47% but this was only measured in women, who are reported to have a lower Hct [5, 9]. The study of Wilhelmi et al. was the only one describing concentrations of vWF (50.00–150.00 IU/dl) and antithrombin. (70.00–120.00 IU/dl) [3]. They observed a fibrinogen concentration of 1.80–3.50 g/l and all other studies had a value within this range [3–6]. Finally, when analyzing clotting tests, we found different results for the PT going from 9–13 s in the study of Foley et al. up to 21 s in the study of Siller-Matula et al. which is around 2 times higher [5, 6]. Gruzdeva et al. reported a value in between (14.2–16 s) with an average of 14.8 s [4]. The aPTT was similar in all studies with Foley et al. describing a value of 24–39 s [5]. The results of Wilhelmi et al. (26–35 s) and Gruzdeva et al. (28.1–30.1 with an average of 29.1 s) were in this range [3, 4].

#### Sheep

Sheep are frequently used as a preclinical animal model for the assessment of cardiovascular device function and biocompatibility. Adult sheep have hearts that are similar in size to the human heart for evaluation of implantable devices [10]. Apart from its advantageous anatomical dimensions, another feature of the ovine organism is that it allows for the evaluation of tissue calcification and thus the conduction of hemo- and biocompatibility studies [3]. It is however reported that sheep have a tendency to lower coagulability [6, 11]. The question remains why?

To study hemo-and biocompatibility of cardiovascular devices, Wilhelmi et al. published a reference list of normal ovine blood parameters relevant to blood coagulation [3]. Blood samples were taken from a cohort of 47 ewes that were all 6 months old. The measured parameters were compared to normal human references. A higher value of platelets was observed in sheep (327,200–550,700/ml vs. 150,000–450,000/ml) and is confirmed in other studies where platelets were 2 to 4 times higher [3, 4, 6]. Fibrinogen (with 1.78–2.15 g/dl in sheep and 180-350 g/dl in humans), vWF (101.5–118.05 IU/dl vs. 50.00–150.00 IU/dl) and aPTT (30.74–35.26 s vs. 26.00–35.00 s) did not significantly differ. The hematocrit (26.0–29.0% vs. 41.5–50.4%), antithrombin (65.77–72.72 IU/dl vs. 70.0–120.0 IU/dl) and prothrombin time (52.26–57.2% vs. 70.00–130.00%) were lower [3]. In contrast to what is generally observed, these values would insinuate a higher coagulability.

Another study comparing sheep and coronary heart disease patients found an antithrombin reduction of 13% and a protein C reduction of 72% in sheep [4]. The antithrombin activity and protein C activity were 1.2 and 3.5 times lower (*p* < 0.05) and the fibrinolytic activity of sheep blood plasma was 60% lower than in CHD patients (*p* < 0.05) [4]. These values indicate a lower fibrinolytic capacity in sheep.

The lower PT observed in the study of Wilhelmi would mean that sheep have a more active external coagulation pathway, which is influenced mainly by factor VII level in the blood plasma [3]. There are studies however reporting prolonged prothrombin time in sheep due to low levels of factor VII. There is some evidence that the level of factor VII is only 13% of the normal values typical for human plasma, other sources report it to be 36 to 45.5% [4]. These results suggest a less active external pathway of coagulation in sheep.

Coagulation dynamics can be assessed using rotation thromboelastometry (ROTEM). This was carried out in the study of Siller-Matula et al. where they compared the coagulation profile of humans, rats, pigs, sheep, and rabbits [6]. Thromboelastography (TEG) does not only quantify platelets and coagulation factors but also examines the dynamics of clot formation. Blood samples of 6 sheep were evaluated. The sheep had four times as many platelets as humans (742 G/l vs. 187 G/l), while again both their baseline fibrinogen level and aPTT were in the normal human range [6]. The α-angle value however, reflecting the increase in clot strength and characterizing functional fibrinogen activity in whole blood, was in the study of Gruzdeva et al. higher in sheep than in CHD patients [4]. The similarity in aPTT found in other studies supports the theory that there are no big differences in the internal coagulation system between sheep and humans [4, 5]. The PT of sheep was twice as high as that of humans (40 s vs. 21 s, *p* < 0.001) whereas the PT of pigs was only a little higher (23 s, *p* < 0.05). This is again contradictory to the result of the study of Wilhelmi et al. and confirms a certain hypocoagulability [6].

When analyzing the clot formation, they found that the clotting time (CT) without thrombin stimulation and the sum of the clotting time and the clot formation time (CT + CFT) were comparable in humans and sheep[6]. A thrombin dose of 0.02 IU decreased the CT by 90% as compared to the control in humans and sheep (*p* < 0.05). In pigs, 0.2 IU of thrombin was required to shorten the CT (*p* < 0.05), which was a 100-fold higher dose of thrombin as compared to humans and sheep [6]. The maximum clot firmness (MCF) of sheep and pigs was in the same range (72–75 mm) but differed from the MCF of humans (58 mm), meaning that both sheep and pigs have a stronger clot formation than humans. A thrombin dose of 0.06 IU caused a 20% decrease in the MCF of humans (*p* < 0.05) and a thrombin dose of 1 IU caused a reduction of 45% (*p* < 0.004). This was in contrast to sheep and pigs where thrombin did not have an effect on the MCF, which may be due to higher thrombin doses required. The median maximum lysis (ML) without thrombin stimulation was similar in humans (21%) and pigs (17%), which was on average nine-fold higher than in sheep (2%) (*p* < 0.001). Thrombin stimulation did not alter the ML in any species. A possible explanation of why thrombin did not alter fibrinolysis in this experiment might be an impaired activity of the thrombin-activable fibrinolysis inhibitor (TAFI) in vitro. The physiologic activator of TAFI is the thrombin-thrombomodulin complex and the concentration of plasma-soluble thrombomodulin in the collected blood is possibly too low for activation of TAFI. This study suggests that sheep could be a suitable species for translational coagulation studies because their clotting time is most similar to humans and that pigs are more useful for the examination of the fibrinolytic pathway [6].

Another study using TEG to evaluate clot formation in 53 sheep (compared with 130 humans) found a significantly shorter reaction time (4.9 min) and significantly higher maximum amplitude (68.6 mm), clot strength (11.9 kd/s), and coagulation index (1.5) [10]. A greater overall clot firmness and a reduced capacity for clot lysis was also observed by Foley et al. [5]. We can draw the conclusion from these results that, once activated, ovine platelets are relatively hypercoagulable with increased platelet function compared to humans [5, 10].

The process of platelet thrombus formation consists of platelet adhesion followed by activation and aggregation. It is obvious that sheep have a higher platelet count but it is not clear whether these platelets are also more active than human ones. One study noticed that ovine platelets adhere and aggregate more strongly to glass compared to human platelets. However, ovine and human platelets differ only slightly in activatability by glass [12]. Gruzdeva et al. discovered that sheep have a significantly higher platelet activation by ADP compared to that of coronary heart disease patients, which is confirmed in other studies [4, 8]. However, this is contradictory to the results in the study of Foley et al. [5]. The obtained result of higher ADP activation can be attributed to the increased number of platelets in the blood plasma of animals, which is accompanied by increased activation of purinergic receptors [4]. The rate of ADP binding to the P2X1 receptor increases, resulting in higher extracellular calcium influx which leads to alteration in platelet shape and aggregation of platelets with each other via the P2Y receptor. Sheep platelets did not react on epinephrine whereas human platelets did. Since epinephrine is a weak inducer, an increased platelet count is likely to result in an increased proportion of non-aggregated platelets and very low aggregometry values, which was demonstrated.

There was no significant difference between the response to collagen in the study of Gruzdeva et al. but other studies showed there is [4, 5, 8]. With 1 μg collagen as an agonist, human (30.93 ± 6.31 Ω) platelets appear to be more responsive than sheep (20.22 ± 7.30 Ω) (*p* < 0.01) [8]. Foley et al. observed a magnitude of collagen induced platelet aggregation that was less than half of that of humans, despite comparable fibrinogen, vWF and platelet count [5].

Goodman et al. did research on how platelets of sheep, pigs and humans interact with low-temperature isotropic pyrolytic carbon (PYC) valve leaflets, polyethylene (PE), silicone rubber (SIL), and Formvar (FVR) [11]. In all respects, the level of response of sheep platelets was considerably less compared to those of humans. Sheep platelets responded with the greatest extent of spreading on PYC, less on PE and SIL, and least on FVR. No fully spread forms were observed on any material, the level of deposition and surface coverage was much lower than in humans and there was much less interaction between platelets. Since human platelets spread extremely extensively on PYC in vitro, and as spreading is part of how human platelets form large thrombi on biomaterials, this may significantly alter the biological response to PYC in vivo. This does not necessarily mean that the sheep is an inappropriate model since non-anticoagulated sheep platelets do form (microscopic) thrombi in vivo on carbon heart valves [13]. It should be recognized that sheep platelets do not attach, spread, and grow thrombi to the same extent and in the same manner as human platelets do [11].

#### Pigs

The use of pigs as a model species for the evaluation of cardiovascular devices is gaining interest. Hence, it is becoming increasingly important to understand how representative the porcine coagulation system is [11]. Unlike sheep, pigs are reported to be hypercoagulable [2].

A list of normal pig blood parameters can be found in Table 1. It is described by Siller-Matula et al. that the platelet count in pigs (430 G/l) is two-fold higher than in humans (187 G/l) (*p* < 0.001) whereas in sheep it can go up to four times the normal human value [6]. This higher platelet count in pigs is confirmed in other studies and may contribute to the higher coagulability [7, 14, 15]. Pigs have just like sheep a similar aPTT compared to human standards [6, 15]. In the study of Siller-Matula et al., pigs had a slightly higher PT (23 in pigs vs. 21 s in humans; *p* < 0.05) which is as previously mentioned also the case for sheep [6].

Fibrinogen levels of the pigs were in the normal range of humans (180–390 mg/dl) [6]. This value of fibrinogen concentration is also seen in the study of Martini et al. where they found a fibrinogen concentration value of 185.8 ± 15.5 mg/dl and in the study of Zentai et al. where a value of 170 ± 47 mg/dl was observed [16, 17]. Martini et al. stated in their study that the control pigs had a hematocrit (Hct) of 29.2 ± 0.7% and a Hct of 32.7 ± 3.2% was seen in the study of Mueller et al. [15, 17]. This is however different from the results in the study of Clauser et al. where pigs had a Hct of 40 ± 3.4% and a normal reference of 31–51% is given. Humans in their study had a Hct of 42 ± 4.0% which is within their reported reference value (37–50%) [7]. Given these values, we can conclude that also the Hct of pigs and humans is approximately equal.

Mizuno et al. analyzed the coagulation dynamics of humans, pigs, calves, and goats with ROTEM and concluded that with the exception of MCF and MCF-*t*, the clotting values of minipigs were the most similar to those of humans [2]. Pigs had an activated clotting time (ACT) of 116.7 ± 10.5 and humans an ACT of 103.1 ± 7.1 s, with no statistical difference. The extrinsic and intrinsic CTs in pigs were not different from those of humans and the α-angle was in the same range. The MCF of the coagulated clot however was higher than that of humans (*p* < 0.01) and the time to MCF (MCF-*t*) was shorter in pigs, indicating faster and stronger platelet aggregation [2].

In the study of Siller-Matula et al., species differences in the coagulation profile with and without thrombin stimulation in vitro were assessed in whole blood. When evaluating coagulation dynamics, they observed that the clotting time without thrombin stimulation in humans (595 s) was 2.5-fold longer than in pigs (244 s) (*p* < 0.05) [6]. In pigs, 0.2 IU of thrombin was required to shorten the CT (*p* < 0.05), which was a 100-fold higher dose of thrombin as compared to humans and sheep where a thrombin dose of 0.002 IU already decreased the CT by 90%. The MCF without thrombin stimulation was in the same range in sheep and pigs (72–75 mm) but was higher than in humans (58 mm), indicating a certain (in vitro) hypercoagulability [6]. A thrombin dose of 0.06 IU caused a 20% decrease in the MCF of humans (*p* < 0.05) and a dose of 1 IU caused a reduction of 45% (*p* = 0.004), whereas thrombin stimulation did not alter the MCF in pigs. A likely explanation for this reduction in humans could be that high doses of thrombin may lead to PLT aggregation and thereby a decrease in PLT counts, which results in a reduction of MCF. In contrast, thrombin did not cause changes in MCF in pigs, which may be due to higher doses of thrombin required since pigs have higher PLT counts [6]. This theory is supported by other studies [18, 19].

The observed median maximum lysis (ML) without thrombin stimulation was similar in humans (21%) and pigs (17%), which was on average nine-fold higher than in sheep (2%) (*p* < 0.001) [6]. Thrombin stimulation did not alter the ML in any species. They found that the endogenous thrombin generation or potential (ETP) of humans (4235 nM·min) and sheep (4092 nM·min) was 50% higher than in pigs (2043 nM·min; *p* = 0.019). However, the lag phase of thrombin generation was more than 90% higher in humans than in any other species (*p* < 0.05). This study confirms the potential usefulness of the pig as an experimental animal species for examining the fibrinolytic pathway, but as previously mentioned it also suggests that sheep could be a suitable species for translational coagulation studies [6].

Just like in sheep, it seems that, once activated, porcine platelets show stronger aggregation than human platelets [2, 6]. It remains of course important to not only evaluate platelet aggregation, but also the activatability of porcine platelets since this determines the strength of clot formation as well. It has been observed in the study of Greif et al. that porcine platelets show no difference in activation by exposure to glass whereas sheep platelets seem to be more activated by glass than human platelets [12, 20].

Clauser et al. studied the differences in PLT activation by collagen (2.5 µg/ml, 5 µg/ml, 10 µg/ml) and ADP (5 µM, 10 µM, 20 µM) between abattoir porcine blood (*n* = 30) and humans (*n* = 30) using light transmission aggregometry (LTA) [7]. They did not find significant differences at concentrations above 10 µM ADP and 5 µg/ml collagen for most parameters. The stimulation of porcine abattoir blood with either ADP or collagen showed considerable activation of platelets. This proves that abattoir blood is not maximally preactivated and therefore gives evidence to the hypothesis that this blood model is suitable for in-vitro thrombogenicity testing in general [7].

The maximum aggregation after collagen activation presented comparable values for human and porcine blood (80%–90%) [7]. The slope of aggregation differed significantly at 2.5 µg/ml and 5 µg/ml collagen (all *p* < 0.01) and the concentration x species effect was rated as significant as well (*p* < 0.01). Lag phases were very similar for both species after collagen activation, becoming lower with increasing collagen concentration. There were no significant differences in disaggregation with and without collagen. Since the slope of aggregation was the only parameter that presented a significant concentration × species effect, they concluded that overall activation with collagen revealed no significant differences in platelet behavior of pigs and humans [7].

In contrast to collagen, stimulation with ADP resulted in a lower maximum aggregation and a significantly higher disaggregation for porcine blood [7]. The maximum aggregation with ADP activation in humans was 63–77% compared to 48–63% in pigs. Of interest, standard deviations were larger for human samples (up to ± 24%) than for porcine (up to ± 10%). The slope of the aggregation curve showed similar behavior with slightly higher values for human blood, and species comparisons revealed significant differences for only 10 µM (*p* = 0.03) and 20 µM (*p* < 0.01), which also applies for the concentration × species effect (*p* < 0.01). The lag phase showed similar behavior for both species, only differing in the reaction with no activation. Consequently, solely inner-species concentration presented sporadic significant differences [7].

The higher disaggregation of up to 70% after ADP activation in pigs reveals that platelet activation is not stable and thus reversible. The same phenomenon has already been reported for pigs as well as other species earlier. Thus, the reversible ADP-activation is a porcine platelet characteristic, which is present in abattoir blood as well, indicating a normal platelet behavior in that special blood model. Despite generally lower values of maximum aggregation in the abattoir blood, no severe differences or malfunctions were obvious for the abattoir blood. These results prove porcine abattoir blood to be comparable to human donor blood at least in terms of ADP and collagen activation [7].

## Discussion

### Sheep

As previously mentioned, sheep are frequently used as a preclinical animal model for the assessment of cardiovascular devices. Adult sheep have a heart that is very similar to the human heart and are therefore well suited for the evaluation of implantable devices [10]. This still has to be done with cautiousness since sheep are of course not exactly the same as humans. An example is the case of the Medtronic Parallel valve, which was tested on sheep and showed little to no thrombogenicity. This led to an FDA approval of the bileaflet mechanical heart valve but when implanted in human patients it did lead to thrombus formation [21]. The failure of this valve prosthesis might have been prevented if the differences in coagulation between sheep and humans were better acknowledged.

The observed results of the sheep coagulation system in this review are somewhat contradictory. Fibrinogen concentration, vWF concentration and aPTT did not significantly differ, indicating no big differences in the internal coagulation system between sheep and humans [3–5]. The hematocrit, antithrombin activity and protein C activity were lower in sheep [3, 4]. Most likely, the decrease in antithrombin III activity in sheep is due to interspecies differences in the substance structure as compared to humans. Human antithrombin and that of sheep are known to be 89% similar in the amino acid sequence. Unlike human antithrombin III consisting of a single polypeptide chain with 432 amino acids, sheep antithrombin III has an additional amino acid at position 6. It is not yet clear what causes the reduction in protein C activity [4]. The PT of sheep was in only one study lower and is generally considered to be higher in sheep [3, 4, 6]. This may be due to low levels of factor VII. There is some evidence that the level of factor VII is only 13% of the normal values typical for human plasma, other sources report it to be 36 to 45.5% [4]. These results suggest a less active external pathway of coagulation in sheep. The lower antithrombin and lower protein C activity indicate a lower fibrinolytic capacity in sheep, but when looking at the coagulation pathway, it seems that sheep are relatively hypocoagulable compared to humans.

Not all results point in the same direction when analyzing ovine platelet characteristics. The clotting time was similar compared to humans but sheep had a 2 to 4 times higher platelet count [3, 4, 6]. Still, sheep could be a suitable species for translational coagulation studies because their clotting time is most similar to humans [6]. The α-angle value, reflecting the increase in clot strength and characterizing functional fibrinogen activity in whole blood, was just like the MCF higher in sheep [5, 10]. We can draw the conclusion from these results that, once activated, ovine platelets are relatively hypercoagulable with increased platelet function compared to humans. Interestingly, it appears that sheep platelets are less activatable than human platelets which might cancel out the platelet hypercoagulability. There were varying results of the activation with ADP, but with collagen were sheep platelets less responsive than human ones [4, 5, 8]. Collagen-induced PLT aggregation is dependent on endogenously generated thromboxane A2. When TXA2 degrades, it forms the inactive metabolite thromboxane B2 (TXB2) [5]. Even though there is a concentration-dependent relationship between collagen concentration and TXB2 release, sheep platelets appear to only release 17.5% of the amount of TXB2 that human platelets secrete for the same collagen stimulus. The contribution of the collagen receptor expression and the mediators of these signaling pathways to the diminished platelet response are yet to be investigated [5]. As long as several platelet receptors are involved in the realization of collagen effects, it can be suggested that one of the pathways promoting aggregation is less prominent in sheep. Supposedly, expression of integrin α2β1 and/ or glycoprotein VI (GPVI) receptors permitting collagen to bind directly to the platelet surface is reduced [4, 8].

Goodman SL. did research on how platelets of sheep, pigs and humans interact with low-temperature isotropic pyrolytic carbon (PYC) valve leaflets, polyethylene (PE), silicone rubber (SIL), and Formvar (FVR). The level of response of sheep platelets was in all materials considerably less compared to those from humans, confirming a certain hypocoagulability.

It is important to notice that from an anatomical point of view, sheep have a very similar heart in comparison to humans, but they are herbivore ruminants (with 4 stomachs) whereas humans and pigs are monogastric omnivores [3, 10, 22, 23]. Ruminants are reported to have a lower susceptibility to anticoagulants, meaning that sheep might not be the best animal for oral anticoagulation testing [22]. This lower susceptibility may be the result of a combination of several factors including the presence of a large forestomach in ruminants (allowing dilution of anticoagulant) and ruminal production of vitamin K1 (which may partially counteract the toxic effects of anticoagulants). Further investigations are required to confirm these hypotheses [22]. Moreover, McKellar et al. and Greiten et al. created a thrombogenic model of the pharmacokinetics of DOACs in pigs, but this does not exist to this day for sheep [24–27]. Further research on the pharmacokinetics of oral anticoagulation in sheep is necessary.

### Pigs

The use of pigs as a model species for the evaluation of cardiovascular devices appears to be increasing [28]. Hence, it is becoming increasingly important to understand how the porcine coagulation system functions. Pigs are reported to be hypercoagulable, which has the advantage of always showing the worst-case scenario since pigs will have more thrombus generation than humans. This can on the other hand lead to the rejection of devices that are actually safe for humans.

Just like in sheep do pigs have similar fibrinogen levels, a comparable aPTT and a slightly higher PT [6, 15–17]. This means that pigs might also have a less active external coagulation pathway with no big differences in the internal coagulation system. The Hct of pigs is approximately equal to the Hct of humans [7].

When evaluating TEG results, a comparable median maximum lysis (ML) without thrombin stimulation was observed in humans and pigs, confirming the potential usefulness of the pig as an experimental animal species for examining the fibrinolytic pathway [6]. The clotting values of minipigs were very similar to those of humans with the exception of MCF and MCF-*t *[2]. This similarity is probably due to the comparable functional structure of coagulation proteins in humans and pigs [2]. The MCF of the coagulated clot however was higher than that of humans (*p* < 0.01) and the time to MCF was shorter in minipigs, indicating faster and stronger platelet aggregation [2, 6]. There might be a slight difference between the coagulation of pigs and minipigs since clotting time without thrombin stimulation in humans was 2.5 fold longer than in pigs (*p* < 0.05) whereas it was comparable to the CT of minipigs [2, 6]. The hypercoagulability in pigs is possibly caused by a higher platelet count compared to humans [6, 7, 14, 15].

When comparing the activity of porcine and human platelets, it appeared that the stimulation of porcine blood with both ADP and collagen showed considerable activation of platelets [7].The maximum aggregation after collagen activation presented comparable values for human and porcine blood (80–90%) [7]. Since the slope of aggregation was the only parameter that presented a significant concentration × species effect, the conclusion could be made that overall activation with collagen revealed no significant differences in the platelet behavior of pigs and humans [7].

In contrast, stimulation with ADP resulted in a lower maximum aggregation and a significantly higher disaggregation for porcine blood [7]. Despite generally lower values of maximum aggregation in the pigs, no severe differences were observed. These results prove porcine blood to be comparable to human donor blood at least in terms of ADP and collagen activation [7]. Because of the similar responses observed in human and pig blood, pigs have been proposed as a suitable model for in vivo evaluation of hemocompatibility of medical devices [2, 11, 29].

## Conclusion

Sheep and pigs are the two most used species in cardiovascular research. However, there are some apparent differences in coagulation between humans, sheep, and pigs. It is clear that the coagulation system of both sheep and pigs still needs exploring since adequate knowledge of these differences is key in the selection of the appropriate species for preclinical cardiovascular research. Future research should compare sheep and pigs in an identical experimental model for thrombogenicity.

## Data Availability

No new data were created or analysed in this study. Data sharing is not applicable to this article.
